# Flexible Wearable Composite Antennas for Global Wireless Communication Systems

**DOI:** 10.3390/s21186083

**Published:** 2021-09-10

**Authors:** Rui Zhang, Jingwen Liu, Yangyang Wang, Zhongbao Luo, Binzhen Zhang, Junping Duan

**Affiliations:** 1 Key Laboratory of Instrumentation Science & Dynamic Measurement, North University of China, Ministry of Education, Taiyuan 030051, China; zhangrui111110@163.com (R.Z.); liujingwenk417@163.com (J.L.); S1806149@st.nuc.edu.cn (Y.W.); S1906095@st.nuc.edu.cn (Z.L.); zhangbinzhen@nuc.edu.cn (B.Z.); 2 School of Instrument and Electronics, North University of China, Taiyuan 030051, China

**Keywords:** flexible composite antenna, wireless communication system, SIR structure, SAR

## Abstract

Although wearable antennas have made great progress in recent years, how to design high-performance antennas suitable for most wireless communication systems has always been the direction of RF workers. In this paper, a new approach for the design and manufacture of a compact, low-profile, broadband, omni-directional and conformal antenna is presented, including the use of a customized flexible dielectric substrate with high permittivity and low loss tangent to realize the compact sensing antenna. Poly-di-methyl-siloxane (PDMS) is doped a certain proportion of aluminum trioxide (Al_2_O_3_) and Poly-tetra-fluoro-ethylene (PTFE) to investigate the effect of dielectric constant and loss tangent. Through a large number of comparative experiments, data on different doping ratios show that the new doped materials are flexible enough to increase dielectric constant, reduce loss tangent and significantly improve the load resistance capacity. The antenna is configured with a multisection microstrip stepped impedance resonator structure (SIR) to expand the bandwidth. The measured reflection return loss (S11) showed an operating frequency band from 0.99 to 9.41 GHz, with a band ratio of 146%. The antenna covers two important frequency bands, 1.71–2.484 GHz (personal communication system and wireless body area network (WBAN) systems) and 5.15–5.825 GHz (wireless local area network-WLAN)]. It also passed the SAR test for human safety. Therefore, the proposed antenna offers a good chance for full coverage of WLAN and large-scale development of wearable products. It also has potential applications in communication systems, wireless energy acquisition systems and other wireless systems.

## 1. Introduction

The rapid development of the field of innovative electronic products has aroused a great deal of interest in the next generation of flexible electronic products, especially in mobile communication terminals and wearable electronic products. In recent years, with the wide application of artificial intelligence products, breakthroughs have been made in the development and manufacture of wearable, sensing, electronic devices [1,2], and the demand for small flexible electronic systems is increasing rapidly. Compared with traditional rigid electronic products, wearable, flexible electronic products are more compact, more flexible, more durable, and reconfigurable and have a wider range of applications [3,4]. Antennas are indispensable and important components of wireless electronic equipment [5]. In fact, antenna design for wireless network communication technology and body local area network application has attracted the attention of the research community [6,7,8], in order to achieve wireless communication with artificial coordinates. All communication signals need to be carried out in the wireless network frequency band, so its importance is self-evident. In recent years, wireless communication has even been involved in the research of biodegradable polymers [9]. In WBAN, there are mainly two types of wearable antennas for wireless data transmission, one is the in-body propagation link, which can maximize the efficient use of the antenna because it sacrifices higher gain [10] in exchange for more useful reception capacity. The other is the off-body propagation using a unidirectional radiation antenna to send body function parameters to an external network for storage, diagnosis and treatment [11]. However, no matter which model is chosen, the selection of materials and manufacturing process are still the key factors to achieve wearable antennas. As we all know, the dielectric substrate will have a far-reaching impact on the structure design, physical size and radiation power of the antenna [12,13,14,15]. In order to make flexible microstrip antennas have better parametric performance and lower loss, a certain proportion of metal oxides and nonmetallic synthetic materials are added to form new polymer materials, and then a processing is carried out. Finally, a flexible dielectric substrate with reliable loss is formed [16]. For example, mixing glass with PDMS to reduce loss of the antenna [17] and using polymers to fabricate transparent-flexible wearable antenna [18].

In recent years, many papers have participated in the improvement of polymers as potential materials for antennas to achieve outstanding functional characteristics in different application fields. For example, Z. Hamouda et al. designed and manufactured a dual-band flexible antenna based on polymer magnetic dielectric media nanocomposite [19]. Fang Xu et al. designed a flexible small antenna with enhanced dielectric properties by diffusing lithium–iron particles in PDMS [20]. The ultrawideband textile antenna of 1.198 GHz–4.055 GHz designed by Xiao you Lin et al. is applied to microwave medical imaging [21]. Luca Catarinucci et al. designed 3D printing molds for ceramic-doped silicone substrates to design flexible and conformal antennas [22]. Yan Yang et al. doped Bi3+ into Ni–Zn ferrite ceramics to adjust the microstructure, magnetic and dielectric properties of the material [23]. M. Samsuzzaman et al. added glass microfibers to the circularly polarized S-band dual-band square patch antenna to reinforce PTFE composite substrates [24]. According to Table 1, it is known that the bandwidth of these antennas is narrow, or the radiation patches loaded with rigid materials as dielectric substrates. Therefore, the above antennas cannot be suitable for wearable function in most wireless communication bands. In this paper, through using doped polymer materials as the dielectric substrate of the flexible antenna, the band width has great expansibility. It also can be used in most wireless communication network frequency bands and the efficiency of the antenna is improved.

The purpose of this paper is to design a conformal, compact, low-profile, broadband antenna covering at least 1–9 GHz effective frequency band for wireless communication systems. The dielectric substrate adopts a flexible tangent polymer PDMS-Al_2_O_3_-PTFE with high dielectric constant and low loss, and the structure adopts an SIR structure, which meets the physical requirements of the antenna. Firstly, the fabrication and testing of flexible dielectric substrate with adjustable parameters formed by Al_2_O_3_, PTFE and PDMS doping are introduced. Secondly, the design process and optimization of a monopole antenna with stepped impedance resonant structure are proposed. Finally, the antenna is attached to the simulated human tissue, and the performance impact of the proposed antenna in the wearable field is evaluated by comparing the simulation results with the experimental results.

## 2. Antenna Design

### 2.1. Synthesis and Manufacturing Process of Composite Materials

**Figure 1:**
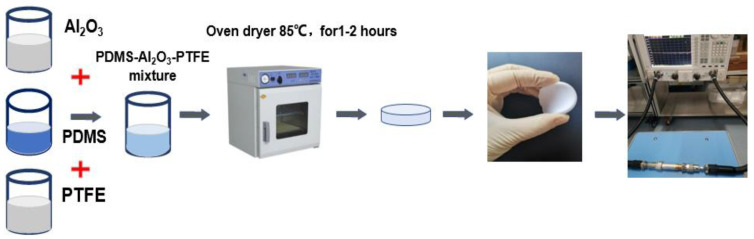
Manufacturing process flow chart.

Polymer matrix composites have attracted wide attention from researchers because of their relatively simple production process and low cost [25,26,27,28,29,30]. PDMS is a kind of flexible organic nonmetallic polymer which is insoluble in water, easily soluble in organic solvents, has high transparency, good chemical inertia, good thermal stability, is homogeneous and is easy to operate. In this paper [31], the equivalent isotropic dielectric constant and loss tangent are verified by two different methods, and the superiority of PDMS in flexible wear-resistant product materials is demonstrated. However, PDMS has low dielectric constant and high loss, which limits its development as a flexible wearable product in the field of wireless communication. One of the main contributions of this paper is the proposal and manufacture of a flexible composite dielectric substrate PDMS-Al_2_O_3_-PTFE with adjustable dielectric constant and loss tangent. The material uses nanoscale Al_2_O_3_ powder and PTFE powder as doping materials. Al_2_O_3_ is chosen because of its high dielectric constant, while PTFE has a lower loss tangent than ordinary material [32,33,34]. Therefore, when trying to dope Al_2_O_3_ and PTFE powder in PDMS, it is necessary to improve the performance parameters and ensure that it is flexible at the same time. Four kinds of composites with different doping rates are prepared and characterized, and the effects of different doping rates on their performance parameters are determined. The samples are established with the PDMS:Al_2_O_3_:PTFE weight ratio of 7.5:2.5:0, 7.5:2.4:0.1, 7.5:2.3:0.2, and 7.5:2.0:0.5. The polymer-based PDMS ratio is preserved at 75% for all samples to maintain the structures flexibility and stability. In the preparation process, firstly, the nanometer Al_2_O_3_ and PTFE are added to the PDMS with an ultrasonic magnetic agitator for a stirring time of more than 400 s, the stirring power is 50%, the pulse power is 40%, and the stirring time is 20 min. For 40 min the mixture is mechanically stirred with a magnetic agitator at the speed of 500 rpm. Then add curing agent and mix it evenly with a magnetic stirrer at the speed of 400 rpm for 20 min. Put it into a vacuum drying oven for 20 min to extract the excess gas and ensure that the oven temperature is consistent with room temperature. The next step is the curing process, the material is placed on the baking table at 85 °C for 1–2 h. Due to the different doping concentrations, the curing time of each group is slightly different. During this period, by observing the curing degree of the material, the curing time is adjusted. The technological process is shown in Figure 1.

**Figure 2:**
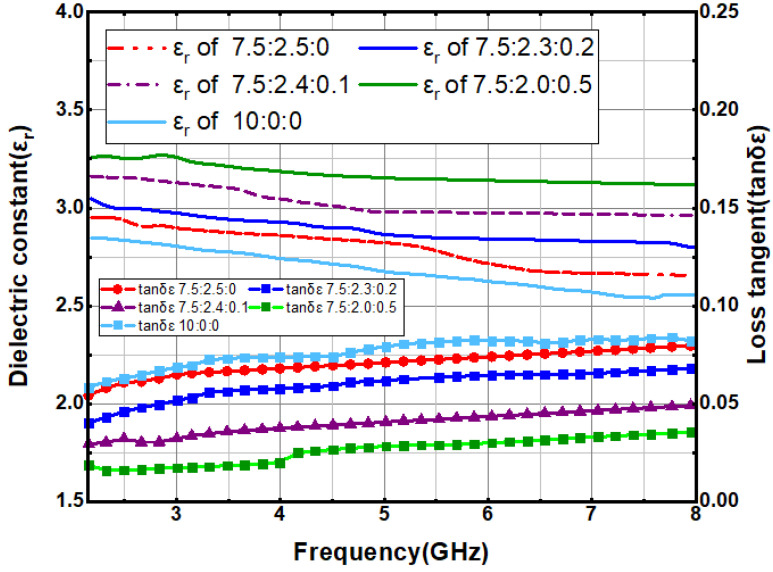
Test results for the dielectric constant and loss tangent of PDMS-Al_2_O_3_-PTFE materials with different doping ratios.

The dielectric constant and loss tangent of the doped materials are tested by a coaxial dielectric probe (Keysight 85054D) and a vector network analyzer. In order to reduce the test error, the coaxial probe is calibrated by standard methods: open circuit, short circuit, and matched load. Figure 2 shows the dielectric constant and loss tangent measurements of four materials with different doping ratios in the range of 2–8 GHz. The test results show that in a certain range, the dielectric constant increases and the loss tangent decreases with the increase of the ratio of Al_2_O_3_ and PTFE. The experimental results show that the dielectric constant and loss tangent will change with the addition of different proportion of doped materials, which can be adjusted to a reasonable range. Finally, the combination of doping ratio 7.5:2.0:0.5 is selected as a new type of dielectric substrate for wearable antenna application.

**Figure 3:**
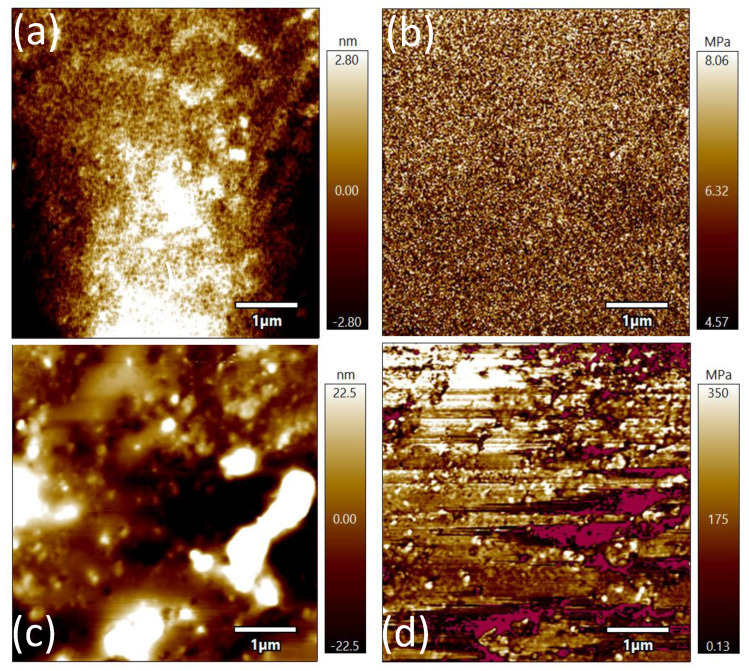
Surface structure of the dielectric substrate (**a**) and the dielectric substrate (**c**) under atomic force microscope. (**b**) Surface structure of the dielectric substrate and Young’s modulus (**d**) under radiation patch and Young’s modulus.

Since the wearable antenna needs to be affected by the external environment, it is necessary to estimate the surface roughness and resistance to load. It can be estimated by sheltering the precise imaging of the Asylum Research Cypher atomic force microscope, which has closed-loop sensors on three scanning axes to ensure high resolution. In the testing process, not only can the atomic resolution be obtained under the closed-loop condition, but also the accurate measurement and positioning at nanometer-level can be obtained to display the two-dimensional morphology information of the synthetic substrate. Figure 3a,c are the surface substrates of composite substrates and micrometric-scale surface roughness of substrates loaded with a metal patch. As can be seen from the surface structure, both of them are smooth. Although the roughness of the radiation patch increases after loading, it is still in the micron order of magnitude and will not affect the stability of its performance. Figure 3b,d show the test data of Young’s modulus. It is known that the Young’s modulus of pure PDMS is 750 KPa, the average Young’s modulus of the polymer composite substrate can reach 6.32 MPa, and the average Young’s modulus after loading radiation metal patch can reach 175 MPa. Therefore, it can better meet the requirements of external load resistance in wearable applications.

According to the above experimental data, after the addition of new materials the dielectric constant and loss tangent of the dielectric substrate change with the different ratios and finally reach the required parameter values. A certain degree of mechanical stiffness is envisaged to guarantee robustness against external value modifications. By maintaining good flexibility, the anti-overload ability of the antenna is also significantly enhanced, and the stability of the use in the external environment is improved.

### 2.2. Structure Design and Theory of Antenna

**Figure 4:**
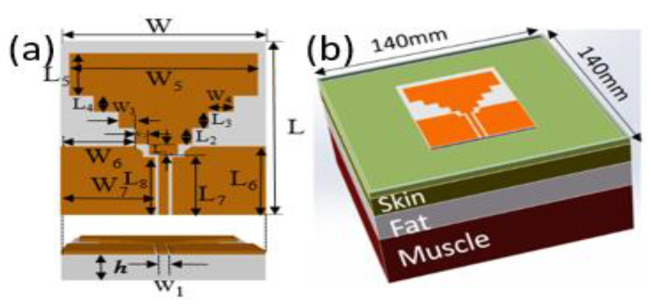
(**a**) Structural Diagram of Antenna (**b**) Construction of Human Tissue.

The antenna is designed and simulated using the finite element software ANSYS High Frequency Structure Simulator (HFSS). The geometry and related parameters of the proposed antenna are shown in Figure 4a, which includes a rectangular radiation patch, a stepped impedance resonator structure, a ground connection, and a 50 Ω coplanar waveguide feed line to obtain a more matching return loss curve. A stepped impedance microstrip structure is adopted between the feed line and the radiating microstrip patch to improve the impedance characteristics of the antenna. In the optimization process, the bandwidth is better expanded by modulating the size parameters of each step of the stepped impedance structure. The rectangular radiating patch is optimized by simulation software. The final optimal structural parameters of the antenna are shown in Table 2.

**Figure 5:**
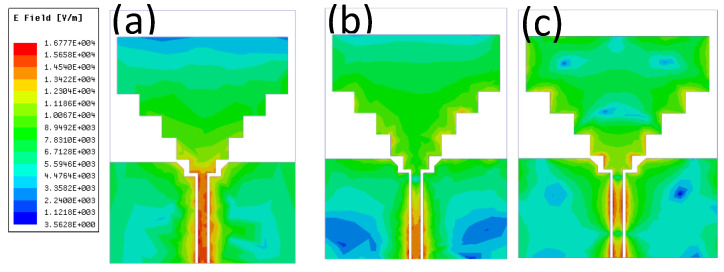
Current distribution diagram (**a**) 2.42 GHz current distribution (**b**) 5.72 GHz current distribution (**c**) 9 GHz current distribution.

The normalized current distribution visually presents the working principle of the antenna. The simulated surface current distribution of the antenna at 2–9 GHz is plotted in Figure 5, respectively. By analyzing diverse current intensity distributions at different frequency points, it is found that the strong current mainly flows on the microstrip line and the edge of the stepped structure. From the observation, it can be seen that even at 9 GHz the overall antenna still has a strong current distribution, which indicates the good overall working performance of the antenna.

For the wearable flexible antenna is designed in this paper, the human body is regarded as the fundamental carrier of the antenna. Discussions also need to be carried out around the functions and requirements of the human body. Therefore, three layers of human tissue are constructed to imitate the structure of human body as shown in Figure 4b. The tissue structure from surface to inside are skin, fat and muscle. Considering the calculation time and accuracy, the distance from the antenna to the outer edge of the tissue structure should be far greater than 1\4 wavelength in the application frequency band. Finally, the skin model size is 140 mm × 140 mm × 30 mm. Table 3 lists the dielectric constants and loss tangent of the body junction at the corresponding frequency band.

**Figure 6:**
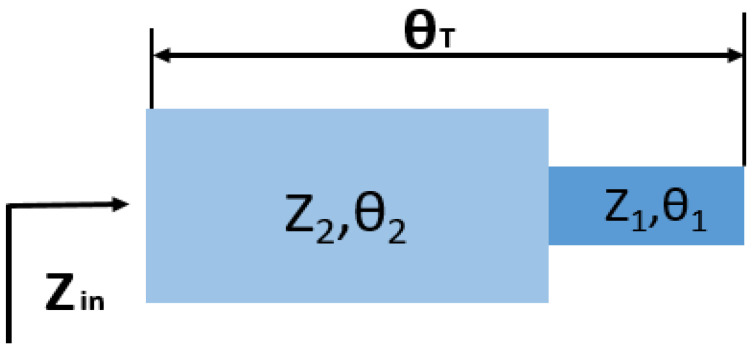
Structure of stepped impedance resonance (SIR).

In this paper, the SIR microstrip resonator is used as the transition structure between the microstrip line and the rectangular radiation patch, which is equivalent to adding a matching network. Adjusting the width of the SIR can effectuate the optimization of impedance matching. By selecting appropriate structural parameter values, a wideband design within a determined range can be realized. Stepped impedance resonator (SIR) structures have been reported many times for design of band pass filters, diplexers and artificial magnetic conductor unit structures. The SIR is a connection of high-impedance and low-impedance transmission lines, and the structure is shown in Figure 6. The high-impedance line acts as a series inductor while the low-impedance line as a shunt capacitor. The SIR resonator can control frequency response characteristics corresponding to the resonant frequency by adjusting the impedance ratio of the resonator and the resonator length to compensate for the uneven velocity of odd- and even-mode phases. Therefore, based on the structure, the wider resonant band can be obtained more conveniently. The two transmission lines of the microstrip have different characteristic impedances (*Z*_1_ and *Z*_2_) and their electrical lengths are *θ*_1_ and *θ*_2_. The two transmission lines of the microstrip also have different characteristic impedances and electrical lengths, respectively. If the effect of step interruption is neglected, the basic resonance condition of SIR can be obtained from the input impedance of the open circuit terminal [35].

(1) $$Z_{in}=jZ_{2}\frac{Z_{2}tg\theta_{2}+Z_{1}tg\theta_{1}}{Z_{2}-Z_{1}tg\theta_{1}tg\theta_{2}},$$

According to the resonance condition *Z_in_* = ∞, the following formula is given.

(2) $$Z_{2}-Z_{1}\;tg\theta_{1}tg\theta_{2}=0$$

The impedance ratio is established:

(3) $$K=\frac{Z_{2}}{Z_{1}}=\;tg\theta_{1}tg\theta_{2}$$

(4) $$tg\theta_{T}=tg\left(\theta_{1}+\theta_{2}\right)=\frac{tg\theta_{1}+tg\theta_{2}}{1-tg\theta_{1}tg\theta_{2}}\;=\frac{1}{1-k}\left(tg\theta_{1}+tg\theta_{2}\right)\;\;\;\;\;=\frac{1}{1-k}\left(tg\theta_{1}+\frac{k}{tg\theta_{1}}\right)$$

when *k* = 1, it can be regarded as a uniform impedance transmission line resonator.

For simplicity, making θ_1_ = θ_2_ = θ_0_, the conditions for fundamental frequency resonance are given.

(5) $$arctg\sqrt{K}=\theta_{0}$$

The spurious frequency is $f_{s}$ and the corresponding θ is θs. From $Z_{\mathrm{in}}=\infty$ and Equation (1)

(6) $$tg\theta_{s}=\infty$$

The spurious resonant frequency $f_{s}$ is as follows:

(7) $$\frac{f_{s}}{\;f_{0}}=\frac{\theta_{s}}{\theta_{0}}$$

Further derivation leads to:

(8) $$f_{s}=\frac{\pi }{2arctg\surd k}f_{0}$$

## 3. Simulation and Test Results

### 3.1. Body Wearablity Test Results 

The applicability of the human wearable devices is further evaluated by numerical simulation and experimental results. When using Agilent N5224A vector network analyzer to measure return loss (S11), the deformation of the flexible antenna will affect the test results, but a complete scan has no effect on the test results. Therefore, the reasonability of the shape and position of the antenna is maintained throughout the scanning cycle to ensure that material deformation has a minimum impact on the results.

**Figure 7:**
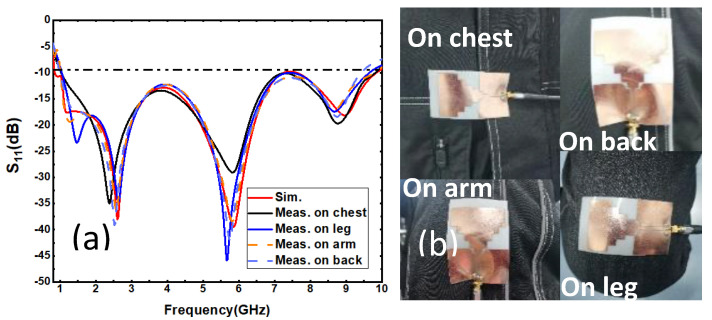
(**a**) Simulation results and S11 test results of different parts of the human body; (**b**) Test pictures of antenna fitting on human body parts.

The measurements on different parts of human chest, thigh, arm and back are in good agreement with the software simulation results. The test results show that the flexible antenna has good adaptability to the human body. As shown in Figure 7, slight, frequency drift and fluctuation are mainly due to the influence of human tissues and fabric of clothes worn by the human body. The average relative impedance bandwidth measured in the human body is 146.7% (1.0–9.4 GHz).

### 3.2. Stability Testing of Conformal Deformation

**Figure 8:**
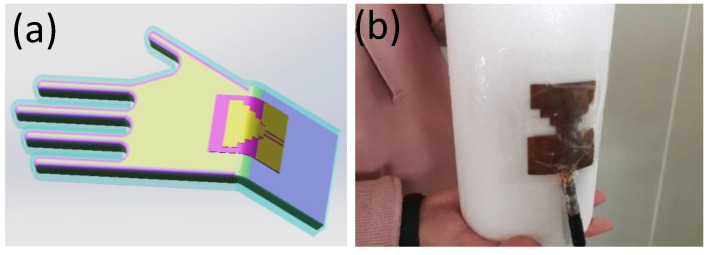
(**a**) Conformal diagram of radial wrist joint and antenna (**b**) Conformal test of antenna.

**Figure 9:**
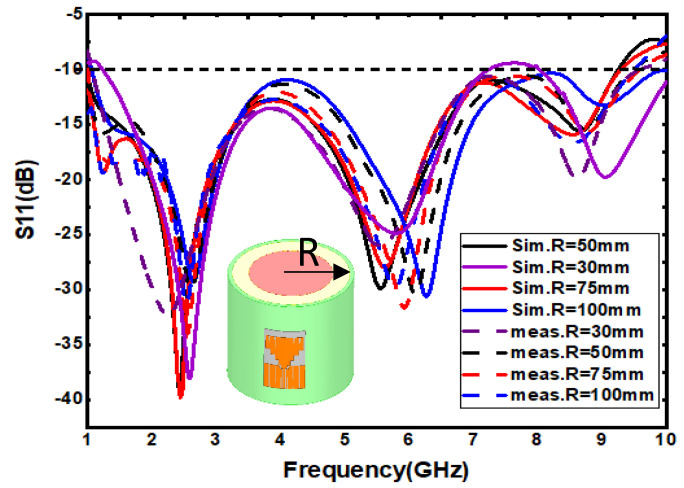
Simulation test results under different radii of curvature.

Due to the uneven shape of the human structure, such as the knee, ankle joint and radial wrist joint, the high degree of deformation is higher when the body is active. Today however, wearable products are more commonly used in the radiocarpal wrist joint and arm and chest. Figure 8a,b are the effects and test patterns of conformal radiocarpal joint and antenna. In order to make the wearable product more comfortable to use, the wearable antenna should be consistent with the uniformity of the deformed state of the human body as far as possible. To verify the consistency of the antenna designed in this paper, four different bending radii of R = 30 mm, 50 mm, 75 mm and 100 mm are used to test the conformal bending of the antenna on behalf of the wrist, arm, leg and chest, respectively. As shown in Figure 9, it can be observed that the measured results of the frequency band are in strong agreement with the simulation results. The measured frequency shift is less than 1.9%, which indicates that the antenna has good robustness. In addition, the radiation patterns under different bending conditions with the same bandwidth are also analyzed. The experimental results show that the radiation of the design is less affected by the structure deformation and is within the error control range.

### 3.3. Safety Assessment of Body-Worn Antenna

In addition to the above confirmatory test, the designer also needs to consider an important safety factor, that is, the proposed antenna will leak electromagnetic radiation into the human body, which will expose the human body to health risks. Therefore, the specific absorption rate (SAR) of the antenna needs to be evaluated to ensure the health and safety of the wearer. A human tissue model is designed in CST, which is composed of skin, fat and muscle, to approach the real human body. The proposed antenna is placed at the height of 3 mm above the simulated tissue. The receiving power of the antenna is set to 0.5 W as a reference. The software simulates the comparison of the SAR data generated by the antenna with the human standard SAR data in band of 2.42 GHz, 5.752 GHz and 9 GHz.

**Figure 10:**
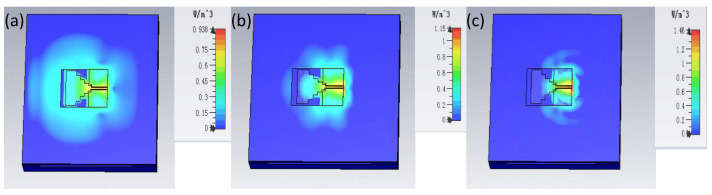
SAR of the proposed antenna distanced by a gap of 3 mm from the human tissue model representing (**a**) 2.42 GHz (**b**) 5.72 GHz (**c**) 9 GHz.

The SAR evaluation is conducted using the IEEE C95.1 standard available in the CST MWS software. The software simulation results are compared with average SAR data and the current distribution, as shown in Figure 10. The results show that the peak SAR results at 2.42, 5.72 and 9 GHz were found to be equal to 0.938, 1.15 and 1.46 W/kg, respectively, which conform to the SAR requirement of lower than 2 W/kg. These results further highlight the superiority of the proposed antenna for on-body applications.

### 3.4. Radiation Pattern Test Results

**Figure 11:**
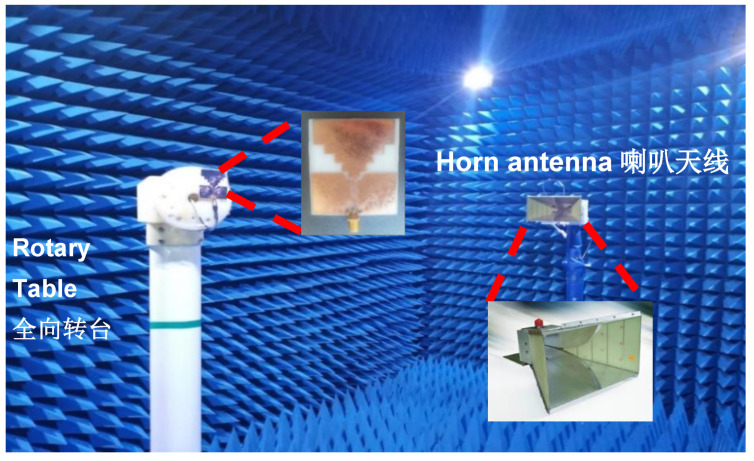
Anechoic chamber antenna physical test diagram.

The radiation pattern results of the antenna need to be tested in an anechoic chamber. As shown in test Figure 11, the transmitting antenna is the standard horn, and the receiving antenna is the tested flexible broadband antenna. The radiation patterns of E and H at different frequency points are tested by rotating the angle of the antenna.

**Figure 12:**
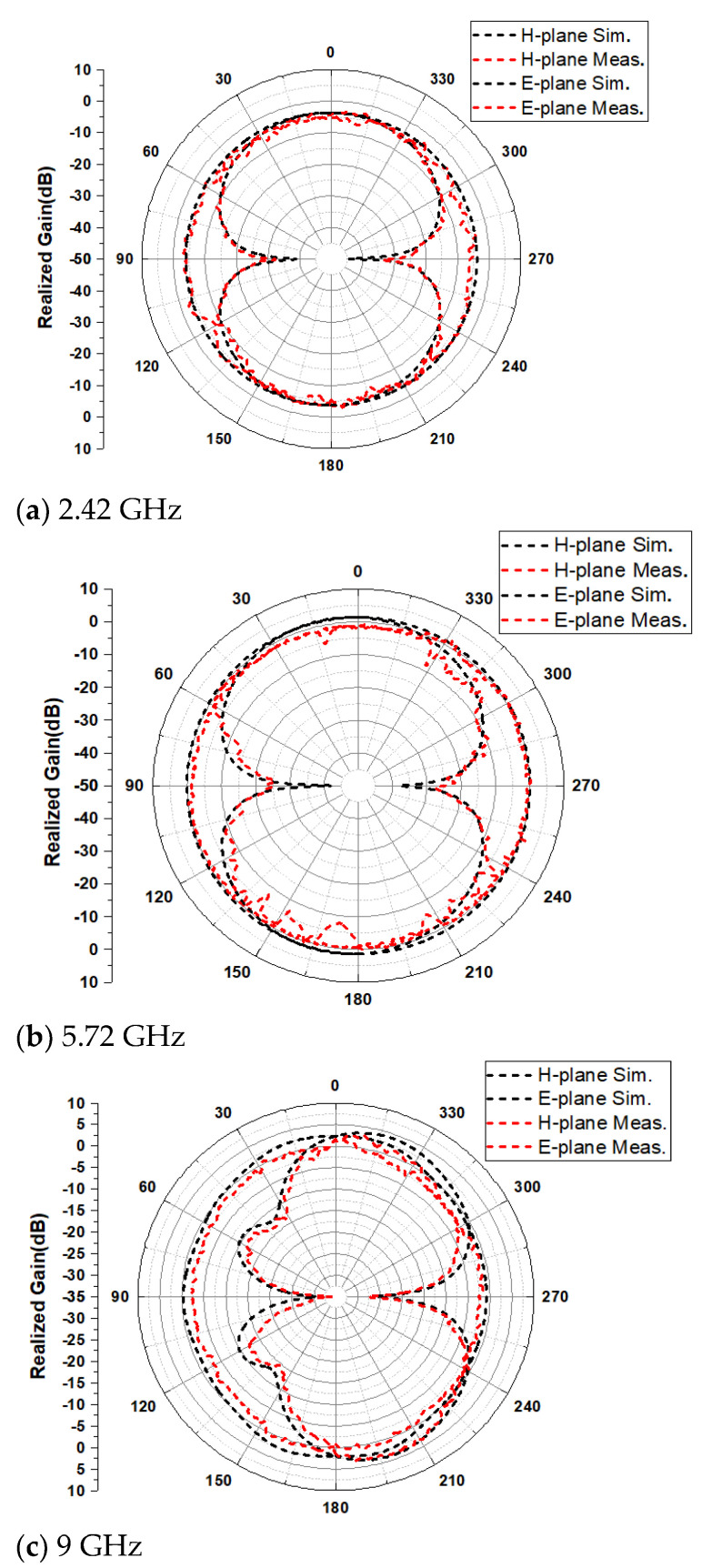
Simulated and measured radiation patterns for the E-plane and H-plane, respectively. (**a**) 2.42 GHz, (**b**) 5.72 GHz, and (**c**) 9 GHz.

**Figure 13:**
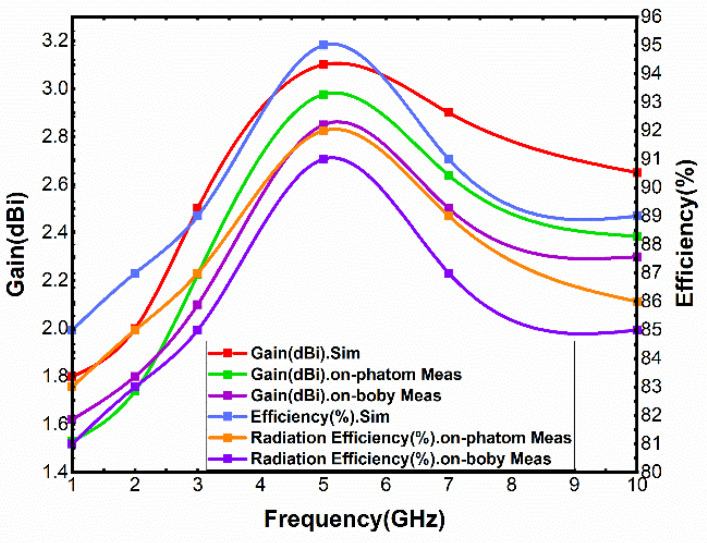
Simulated and measured gain and efficiency *conditions*.

Figure 12 shows the simulation and measurement results of E-plane and H-plane radiation patterns of far-filed radiation at 2.24 GHz, 5.72 GHz and 9 GHz. The results show that there is no obvious distortion on the E-plane and H-plane at the three frequency points. The E-plane is a directional radiation pattern, and the H-plane is close to the omnidirectional radiation pattern. The peak gain is stable at 2–3 dBi. Simulated and measured results of gain and radiation efficiency are presented in Figure 13. The results show that the maximum gain and radiation efficiency are 3.1 dBi and 95%, respectively. In addition, radiation efficiency of more than 83% is obtained for the entire working BW. Once the radiation characteristics of the antenna are investigated and evaluated under test conditions, the capability of the antenna in wearable applications should be evaluated.

## 4. Conclusions

A broadband antenna loaded onto a flexible composite dielectric substrate is presented, which can be used in wireless communication systems. Through the innovative design of the composite dielectric substrate with adjustable parameters, the loss of the antenna is reduced, and the stability of the flexible antenna is improved. The antenna adopts a step impedance excess structure to realize omnidirectional radiation in the range of broadband communication in vivo and in vitro. The working mechanism of the antenna is analyzed, and the effects of structural deformation and human body load on the performance of the antenna are studied. The conformal model is established and measured in different scenes to verify the proposed design scheme. The measured results are on the whole consistent with the simulation results. In addition, SAR simulation results show that the antenna meets the health and safety requirements. Overall, the antenna has the advantages of flexibility, broadband and rugged performance, making it a potential candidate for multifunction wearable devices.

## Figures and Tables

**Table 1:** A comparison of proposed antennas with recent works.

Ref.	Bandwidth	Gain	Composite Material?	Flexible?
[19]	1.75–3 4.6–6.7	2 dBi	Yes	No
[20]	0.1–1.0	-	Yes	Yes
[21]	1.198–4.055	2.9 dBi	No	Yes
[22]	2.35–2.45	2 dBi	Yes	Yes
[23]	0.1–1.0	-	Yes	No
[24]	2.16–2.19 3.29–3.33	5.52 dBi	Yes	No
This work	0.99–9.41	2 dBi–3 dBi	Yes	Yes

**Table 2:** Structural dimensions of the antenna.

Parameter	Value (mm)	Parameter	Value (mm)
L	70	W	50
L1	3.8	W1	2.5
L2	6	W2	3.5
L3	6	W3	3.5
L4	6	W4	3.5
L5	15.8	W5	46
L6	25	W6	18
L7	28	W7	23
L8	24	h	0.5

**Table 3:** Dielectric properties and thickness of human tissue.

Tissue	1.0 GHz–9.5 GHz		Thickness (mm)
	ε_r_	σ	
Skin	39.2–31.0	0.8–5.6	1.5
Fat	5.3–4.2	0.04–0.52	8.5
Muscle	53.0–44.0	0.8–7.9	18

## Data Availability

Not applicable.
